# Design of a novel tendon-driven manipulator structure based on monolithic compliant rolling-contact joint for minimally invasive surgery

**DOI:** 10.1007/s11548-021-02442-w

**Published:** 2021-07-07

**Authors:** Dingzhi Zhang, Yilun Sun, Tim C. Lueth

**Affiliations:** Institute of Micro Technology and Medical Device Technology, Technical University of Munich, Boltzmannstraße 15, 85748 Garching, Germany

**Keywords:** Minimally invasive, Contact rolling joint, Tendon driven, Mechanism design, 3D printing

## Abstract

**Purpose:**

Compliant mechanisms are commonly used in the design of manipulator and surgical robotic tools for minimally invasive surgery (MIS) thanks to their compactness, ability of miniaturization and lower part count. However, conventional compliant joint has higher internal stiffness, which limits the bending radius. To overcome this problem, a novel tendon-driven manipulator structure based on monolithic compliant rolling-contact joint (CRCJ) is proposed.

**Methods:**

The proposed rolling-contact mechanism is used to prevent cable slack during actuation, which occurs in conventional compliant joint design. By means of selective laser sintering (SLS) technique, the CRCJ can be fabricated in a monolithic structure, thus granting the CRCJ both the advantages of compliant joints and rolling-contact mechanism. Simulations with nonlinear finite element analysis (FEA) and experiments were conducted to evaluate and compare the mechanical properties of the proposed CRCJ with conventional leaf-type compliant joint including the bending and compliant motion.

**Results:**

Experimental results showed that the CRCJ has lower bending stiffness, higher maximum bending angle (over $180^{\circ }$) and a higher compliance compared to conventional compliant hinges, which allows a larger workspace and reduces the possibility of tissue injury. Agreement was also found between the nonlinear FEA and experiments regarding the relation between actuation force and bending angle. A primary prototype of a 3-DOF handheld laparoscopic manipulator with a diameter of 7 mm was further developed.

**Conclusion:**

A dexterous tendon-driven monolithic manipulator structure based on CRCJ for MIS is proposed. A preliminary prototype of a handheld laparoscopic manipulator demonstrates the capability of the CRCJ for steerable medical devices. However, design improvements based on FEA and application-orientated prototypes considering anatomical requirements still show room for improvements.

**Supplementary Information:**

The online version supplementary material available at 10.1007/s11548-021-02442-w.

## Introduction

**Fig. 1:**
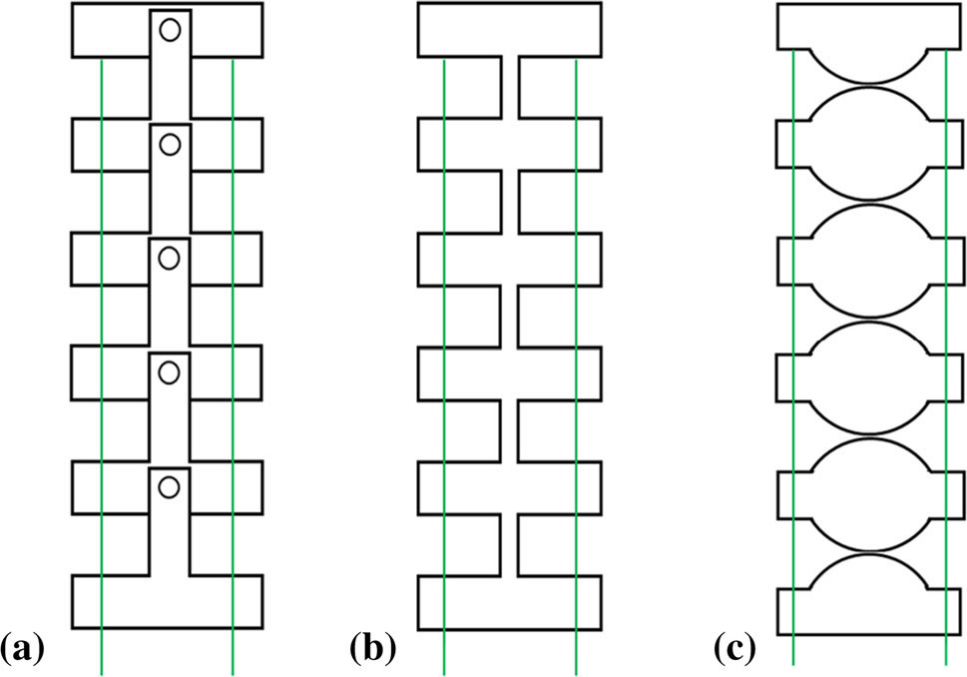
Concepts of joint design for tendon-driven manipulator. **a** Discrete pin joint. **b** Monolithic compliant joint (leaf type). **c** Discrete joint with rolling-contact mechanism

Since the first laparoscopic cholecystectomy back in 1985 by the German surgeon Prof. Dr. Med Erich Mühe, the laparoscopic surgery and therefore also the minimally invasive surgery (MIS) have been continuously improved, which benefits both the patient and physicians in terms of reduced intra-operative trauma, patient’s healing time as well as shorter hospital stay and therefore lower cost [1]. In the initial phases, surgical tools for MIS such as laparoscopic and endoscopic tools consist of rigid or flexible rods. In recent years, MIS of numerous disciplines makes use of hyper-redundant and continuum manipulator [2]. Joint structures have been integrated in the design of the endoscopes and other surgical instruments to make part of the device steerable so that more dexterous motions and new surgical procedures are possible. While pneumatic chambers [3, 4] and hydraulic forces [5, 6] are studied and employed as actuation of continuum robot, tendon-driven mechanism is still commonly used to drive flexible tools for medical applications, that is also applied in the proposed manipulator structure in this paper. With respect to the joint design, flexible joints can be classified into two categories: discrete joint and monolithic structure. Figure 1a, b gives a schematic view of the conventional design of these two structures. The first group of flexible joints is comprised of a series of discrete, rigid links. Tendon is threaded through all rigid links for actuation. The use of conventional pin joint, as shown in Fig. 1a, can be found in the design of MIS surgical instruments. An apparatus for transluminal gastrointestinal procedures was developed by Saadat and Peh [7] containing a flexible and shape-lockable guide with 6 pin joints before the end effector. Pin joints are linked rotated $90^{\circ }$ around the axis successively in [8], forming a flexible bent shaft connecting to the end effector of a handheld MIS instrument. Since the joints are fabricated separately, the assembly process can be time-consuming considering the part count and the small part size.

In contrast to discrete joints, compliant joints are the second category, which can be fabricated monolithically. Due to their monolithic structure and intrinsic elasticity, compliant joints are able to bend continuously, thus can also be denoted as continuum joints. While this kind of joints can also be fabricated using conventional reductive manufacturing techniques such as laser cutting and computer numerical control (CNC) milling, the development of additive manufacturing techniques has promoted the research on compliant joints in medical fields. A manipulator system including an overtube structure for a standard endoscope with two manipulator arms and a mechanical control unit were fabricated using selective laser sintering (SLS) techniques with polyamide in [9], in which the snake-like arms make use of conventional compliant joints (leaf type) consisting of a thin flexible part between rigid sections, similar to Fig. 1b. A long-term stability of this system was evaluated in [10], showing the suitability for use as disposable surgical manipulator system. Feng et al. developed a spring-like flexible joint which was manufactured using selective laser melting (SLM) technology [11]. A metallic 3D printed flexible joint with helical spring-like structure was proposed by Hu et al. [12]. In comparison to [10], a series of circular rolling contacts are introduced at each turn of the helix to reduce axial compression [12]. However, these compliant joints and the conventional discrete joints described previously have the property of unbalanced contour length variation between the agonist and antagonist side under actuation, which results in cable slack and affects positioning accuracy [13].

To cope with this problem, rolling-contact joint can be utilized, which is schematically shown in Fig. 1c. Suh et al. [13] proposed hyper-redundant rolling joints linked by NiTi elastic fixtures, which ensures a stable rolling motion. In [14], Kim et al. removed the elastic fixture by positioning the driving cables in the middle of the discrete rolling-contact joints. Principle of rolling-contact mechanism was also applied in [15] by using protuberance and groove as constrain to ensure pure rolling motion between joints. However, all these applications using rolling-contact joint have the drawback of large part count and thus the assembly effort is relatively high.

In this paper, we introduce a monolithic compliant rolling-contact joint (CRCJ) based on the concept in [16], in which a rolling-contact joint and its crossways arranged flexible bands are in one single part. With the monolithic design, the manipulator structure combines both the advantages of compliant mechanism and the rolling-contact mechanism. The proposed structure can be fabricated using additive manufacturing technique which reduces the part count significantly thus alleviate the assembly process.

The remainder of this paper is structured as follows. Section II introduces the mechanical structure, geometry creation and the fabrication method of the flexible joint. In Section III, the finite element (FEM) model of the proposed structure is first introduced. For the force modelling of the cable-driven mechanism, methods from [17, 18] were applied. Experiments were conducted to compare the mechanical properties of CRCJ with conventional leaf-type joint. In Section IV, a handheld manipulator is demonstrated as an example of medical application. Section V and Section VI provide a discussion, conclusion and outlook of the current work.

## Methods

**Fig. 2:**
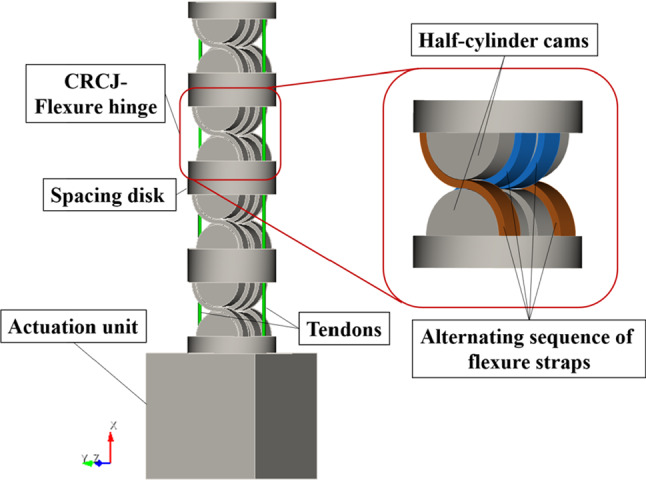
Structure of the CRCJ manipulator section

**Fig. 3:**
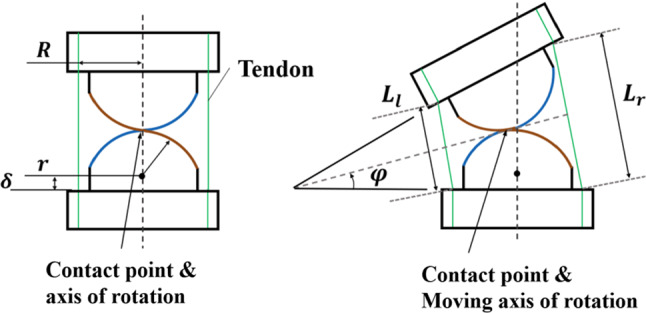
Schematics of the tendon-driven CRCJ. Under actuation, the CRCJ has a moving axis of rotation, which is the contact point of two cams

### Mechanical structure

The soft robot structure is based on CRCJ, which is a compliant joint with rolling contacts. In comparison to continuum manipulators with discrete links using pin joints or monolithic structure using conventional flexure hinges, this rolling motion is able to prevent cable slack during actuation.

The structure of the proposed mechanism is illustrated in Fig. 2. The continuum manipulator consists of flexure hinges with CRCJ aligned in series, separated by the spacing disc. Each enables a plane rolling motion. In order to achieve manipulation in 3-dimensional space, CRCJs in different rolling planes can be arranged in series. A tendon-driven mechanism is utilized for the actuation. For the current implementation, a tendon pair is applied for the rolling motion in each plane.

The CRCJ is composed of two cylinder cams which are joined together by four thin flexure straps in alternating sequence. These straps allow rolling contact between the cams and avoid sliding. Unlike revolute joints, CRCJ has a moving axis of rotation, which is always coincided with the contact point between the cams, as shown in Fig. 3. Compared to conventional flexure hinge without rolling contact (e.g. leaf type), CRCJ can achieve wider range of motion since the rolling-contact mechanism causes less stress and strain energy by deformation. The deformation of a general CRCJ after actuating is illustrated in Fig. 3. It rotates towards the left due to the agonist tendon on the left. The radius of the cylinder cam, the distance between spacing disc and the centre point of cylinder cam, the rotational angle are denoted as *r*, $\delta$ and $\phi$, respectively. Assumed that the CRCJ has a symmetric configuration, *R* denotes the distance between the tendon and the centre of CRCJ. Using geometrical information, the length of the agonist tendon ${L}_{l}$ can be formulated as

1 $$\begin{aligned} L_l = 2(r +\delta \cos \varphi - R \sin \varphi ) \end{aligned}$$

and the length of the antagonist tendon L$_{r}$ is obtained as

2 $$\begin{aligned} L_r = 2(r +\delta \cos \varphi + R \sin \varphi ). \end{aligned}$$

The total length of both tendons is calculated as follows:

3 $$\begin{aligned} L_{{ total}} = 4(r +\delta \cos \varphi ). \end{aligned}$$

In case the term $\delta \cos \phi$ vanishes, the sum of the two tendons length $L_{\mathrm{total}}$ is constant at zero. In our proposed CRCJ mechanism, half-cylinder cams are utilized, which leads to $\delta = 0$. Thus, the sum of the length of the agonist and antagonist tendon of each CRCJ remains the same during actuation. Consequently, the entire tendon length remains unchanged and there is no play in the tendon during actuation and the torque generated from the actuation unit can be transmitted to the tip [19].

### Geometry generation and fabrication

**Fig. 4:**
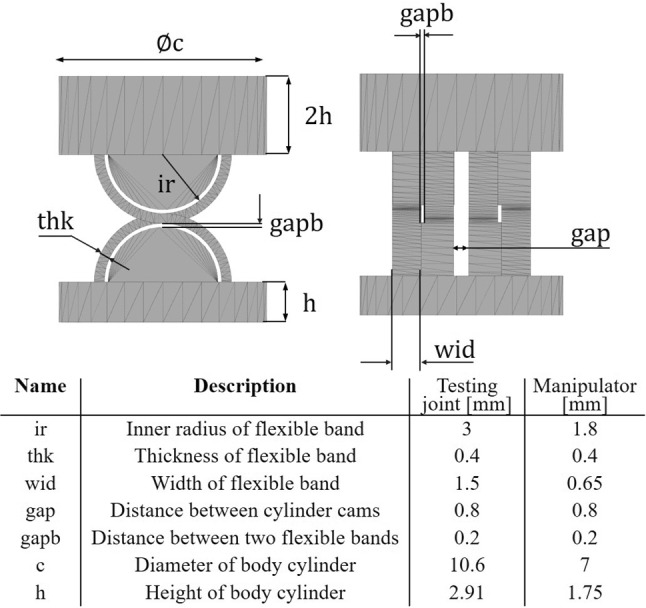
Schematic surface model and design parameters of the proposed CRCJ sections and the parameter values used for the design of testing unit and manipulator structure

The design of mechanism is conducted using the in-house surface modelling language SGCL based on the SG-Library developed by Lueth [20], which is a surface modelling toolbox optimized for additive manufacturing written in MATLAB. A more detailed description of the usage and the modelling process is provided in [18, 21]. Since SGCL is a code-based modelling language, all structural parameters can be parameterized so that the proposed CRCJ structure can be scaled, adjusted and integrated in different prototypes easily. All structural parameters and their description are shown in Fig. 4. The distance *gapb* is left intentionally to compensate the accuracy of the fabrication method. Parameters *thk* and *gapb* are chosen based on our experience with the SLS technology so that the flexibility and the stability of the flexure hinges can be ensured. The parameter *gapb* is chosen the same to the value of 0.8 mm since the same tendon is applied for both the testing joints and the manipulator sections.

The testing units and prototypes using CRCJs within the present work are manufactured with Formiga P100 from EOS$\textregistered$ (EOS Gmbh Electro Optical Systems, Krailing, Germany). The machine is based on the SLS method, which is a powder-based generative manufacturing process. The applied material is polyamide PA2200 [22], which is approved as bio-compatible according to EN ISO 10993-1. Mechanical properties of this material with the test standard ISO 527 that are relevant for FEA are summarized in Table 1.

**Table 1:** Mechanical properties of PA2200

Mechanical properties	Value	Unit
Tensile modulus	1700	MPa
Tensile strength	50	MPa
Strain at break	0.2	–

## Simulation and experiments

### Finite element analysis

**Fig. 5:**
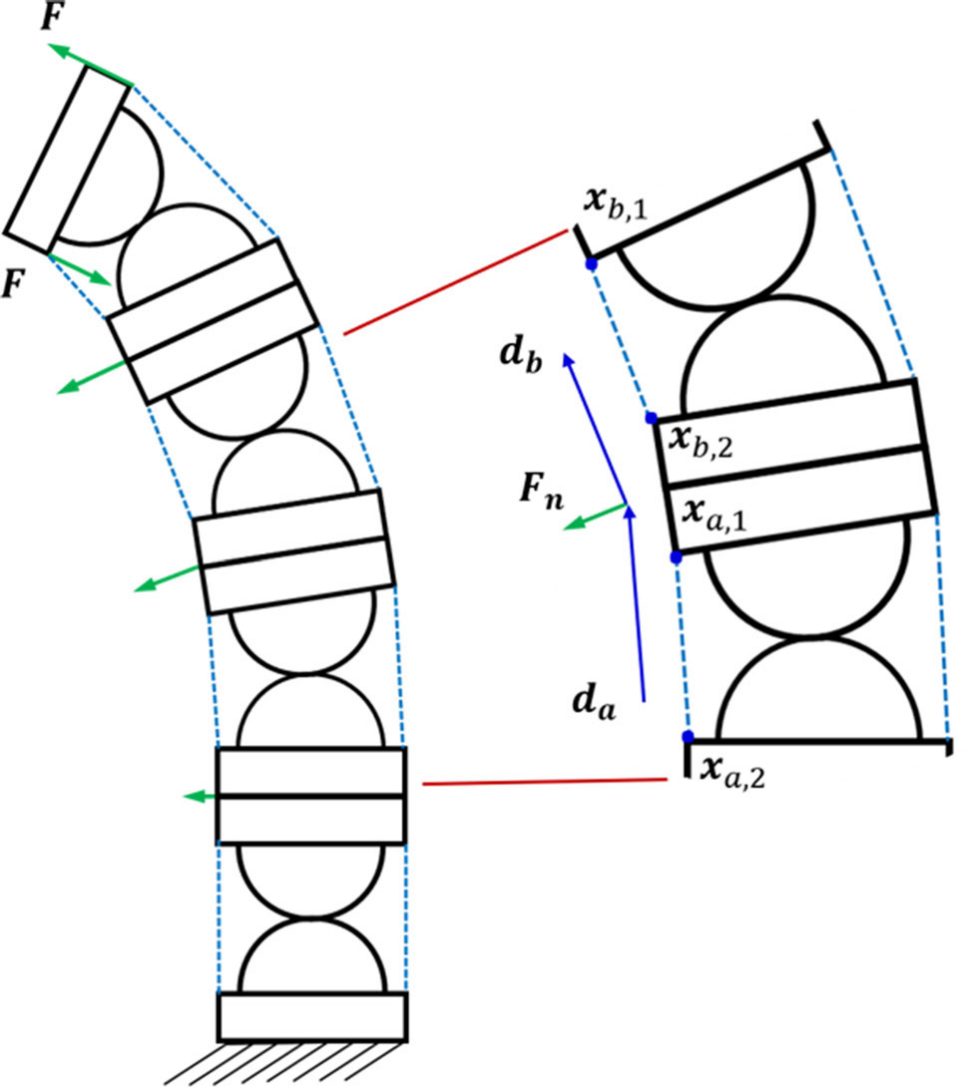
Boundary conditions of the FEM model and the modelling of tendon forces of the CRCJ. The bottom surface of the CRCJ is fixed. External forces acting on the CRCJ include the actuation modelled as force couple $\mathbf{F}$ on the top and normal forces $\mathbf {F}_n$ to the cylinder bodies

Compliant mechanisms are characterized by complex force-motion behaviour due to their compliant nature. Conventional methods such as the pseudo-rigid-body-model (PRBM) offers an approach for the analysis of compliant mechanisms with a limited sense. Mechanisms with complex geometry such as flexure hinges are not able to be precisely modelled using this method [18, 23]. To overcome this problem, a more general approach for the analysis of compliant mechanisms based on nonlinear FEM is implemented in the present work. The FEA of a section of four CRCJs as shown in Fig. 2 is performed by the open-source 3D structural analysis program CalculiX (Dhondt, Groebenzell, Germany). For the mesh generation from surface data of the model, CUBIT (Sandia National Laboratories, National Technology and Engineering Solutions of Sandia, LLC., USA) is utilized. A total of 114,537 C3D4 elements are used to create the FEM model. In addition to geometrical nonlinear effects, contact problems between flexible straps and cams as well as nonlinear cable actuation forces are also considered. Penalty contact with the segment-to-segment approach is applied. As for the modelling of nonlinear tendon forces, methods described in [17, 18] are implemented, which also take into account the normal forces acting on the spacing discs. The boundary conditions of the FEA is schematically illustrated in Fig. 5. Tendon actuation forces are modelled as a force couple resulting in a torque on the tip, which is always perpendicular to the top surface. The direction of the normal forces $\mathbf {d}_{\mathbf{b}}$ and $\mathbf {d}_{\mathbf{a}}$ are dependent on the tendons before and after the tendon guides and can be determined using the current position of the nodes $\mathbf {x}_{\mathbf{b},\mathbf{1}}$ $\mathbf {x}_{\mathbf{b},\mathbf{2}}\ \mathbf {x}_{\mathbf{a},\mathbf{1}}$ and $\mathbf {x}_{\mathbf{a},\mathbf{2}}$:

4 $$\begin{aligned} \mathbf {d}_{\mathbf{b}} = \frac{\mathbf {x}_{\mathbf{b},\mathbf{1}}-\mathbf {x}_{\mathbf{b},\mathbf{2}}}{|\mathbf {x}_{\mathbf{b},\mathbf{1}}- \mathbf {x}_{\mathbf{b},\mathbf{2}}|} \; {\hbox {and}}\; \mathbf {d}_{\mathbf{a}} = \frac{\mathbf {x}_{\mathbf{a},\mathbf{1}}-\mathbf {x}_{\mathbf{a},\mathbf{2}}}{|\mathbf {x}_{\mathbf{a},\mathbf{1}}-\mathbf {x}_{\mathbf{a},\mathbf{2}}|}. \end{aligned}$$

The normal force ${F}_{\mathrm{n}}$ is then calculated as:

5 $$\begin{aligned} \mathbf {F}_{\mathbf{n}} = (\mathbf {d}_{\mathbf{b}}+\mathbf {d}_{\mathbf{a}})F. \end{aligned}$$

**Fig. 6:**
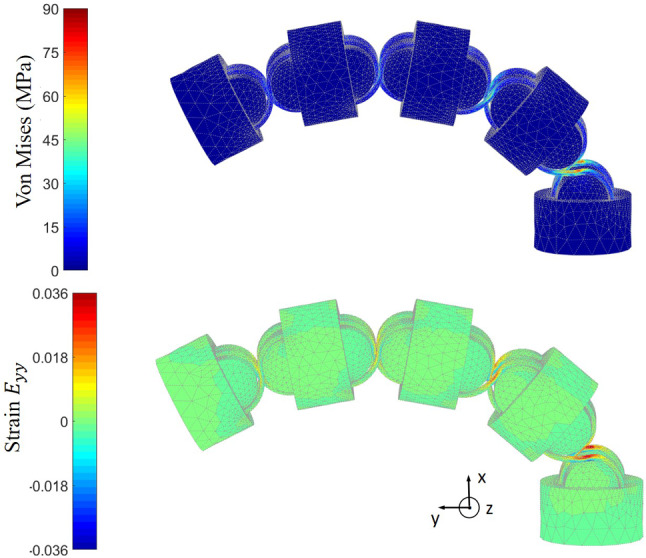
Von Mises stress and normal strain in y-axis direction of the FEA simulation of CRCJ manipulator section with an external load of 12.6 N mm on the top surface

From the stress plot in Fig. 6, it can be noticed that the mechanical stress is mostly concentrated in flexure straps of the CRCJs, especially in the contact regions. The peak von Mises stress in these regions surpasses the tensile strength of the applied material, which is at 50 MPa. This indicates that material yielding may be present. However, one possible reason for the high stress could be shear locking. Linear C3D4 elements are applied to this FEM model, which can produce parasitic stresses under bending. Another reason could lie in the material modelling. For this FEA, the material is modelled as an isotropic linear elastic material using the Young’s modulus given in Table 1. Since the fabricated CRCJ can still return into its initial form after actuation forces are removed, the true material behaviour could be nonlinear elastic due to the imperfections in the fabrication process of SLS. On the other side, the cylinder bodies and cylinder cams are barely loaded and the von Mises stress is close to zero. Similarly, the normal strain in y-direction is also concentrated in the contact region of the flexure straps in the first segment. The maximal absolute value of the normal strain is 5.67 %, which is much smaller than the strain at break of the applied material PA2200 given in Table 1. This shows that the manipulator with the current design is capable of a greater bending until material fails.

**Fig. 7:**
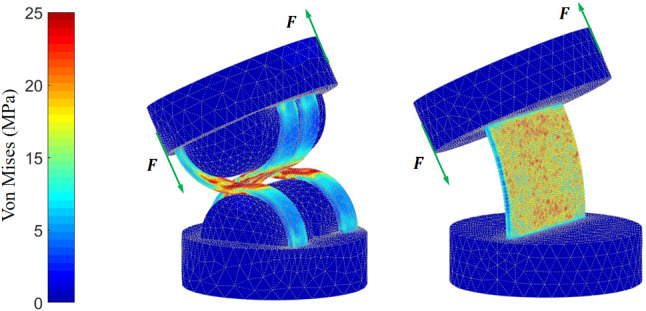
Von Mises stress of the FEA simulation of a single CRCJ section and a leaf-type hinge with same bending angle of $26^{\circ }$

Moreover, FEA for a single CRCJ segment and a leaf-type hinge is conducted to compare the stress occurring in the flexure hinges under same bending angles of $26^{\circ }$. The von Mises stress distribution is given in Fig. 7. While the stress in CRCJ is mainly concentrated in the rolling-contact regions and the rest of the flexible straps remain mostly unloaded, the stress is distributed over the whole hinge area of leaf-type hinge. Since the CRCJ has a moving axis of rotation, which leads to a continuous changing of contact points, there is always a small area of the hinge under load. This can ensure a higher mechanical stability and the fatigue strength of CRCJ compared to conventional leaf-type hinges.

**Fig. 8:**
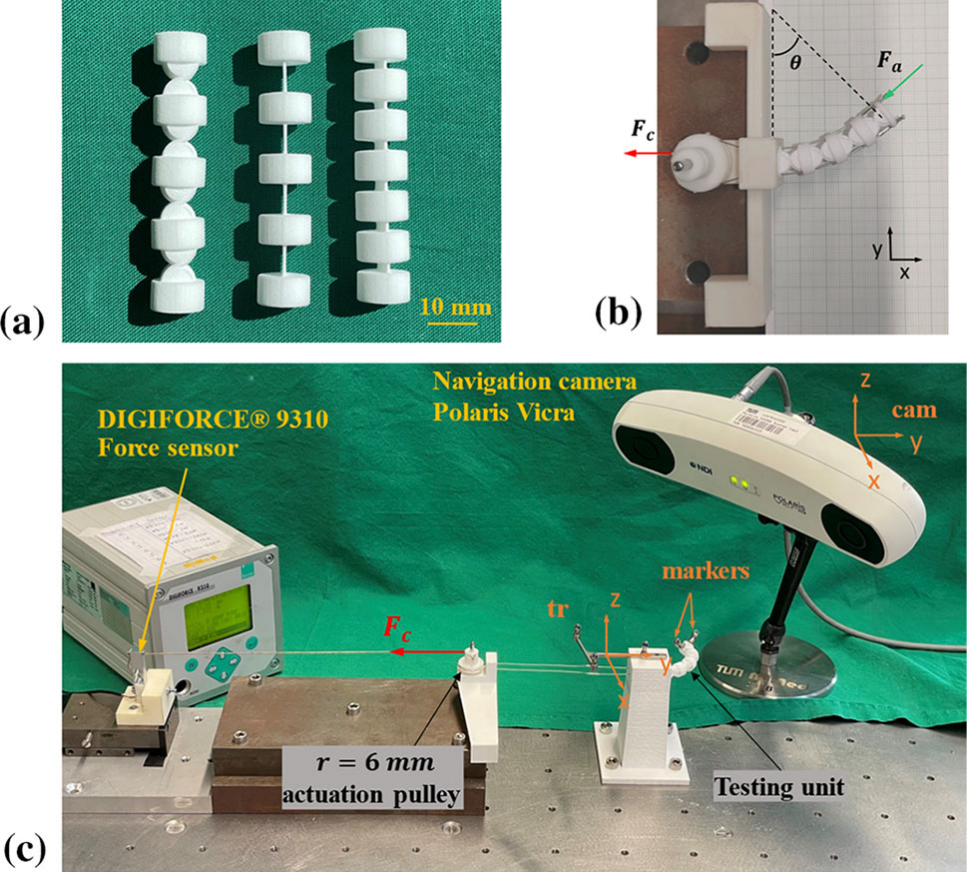
Testing joints and experiment set-up for bending motion test. **a** Testing joints: CRCJ (left), leaf-type 1 (mid) and leaf-type 2 (right) and **b** schematics of the bending motion test. Bending angle $\theta$ and actuation cable force $F_{\mathrm{c}}$ are given accordingly. The threshold of test break is set at $\theta =180^{\circ }$. The bending motion test of leaf-type 2 stopped due to geometric constrain of spacing discs. **c** Experiment set-up of bending motion test

**Fig. 9:**
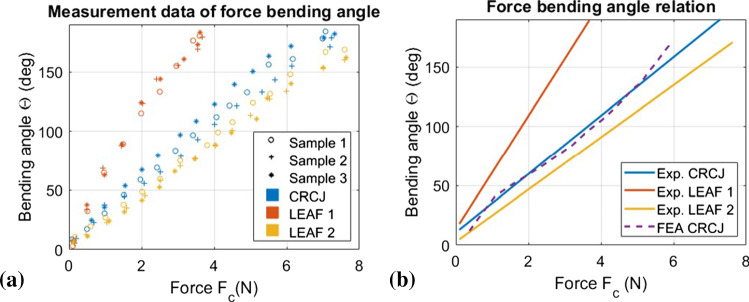
Results of Experiment 1: Bending motion **a** Experiment measurement data of force bending angle relationship of all 9 samples. Linear correlation in all measurement series was identified. **b** The force bending angle relationship from linear fitting of experimental measurement and FEA. For FEA, the correction factor $\kappa =2.13$ was determined

### Experiments

In this section, experiments on testing units were conducted to analyse the bending motion with regard to the actuation force as well as the compliance transverse to the bending plane and in the bending plane (compliant motion) between CRCJs and two conventional leaf-type joints, as shown in Fig. 8a. For the first experiment, 3 samples of each joint type were used. For the second and third experiment, one sample per joint type was measured. The diameter of the spacing discs in all tree joint structures are $c = 10.6\, \hbox {mm}$. The structural parameters of the CRCJ were defined such that the thickness of the flexible straps is the same as the thickness of the leaf-type hinges, which is 0.4 mm. All testing joints have the same length of 54.7 mm. The choices of the leaf-type joints for the comparison are decided considering the following aspects: (1) The first leaf-type joint (cf. Fig. 8a, mid) has the same number of hinges as the CRCJ. (2) To increase comparability of the experiments, the second leaf-type structure (cf. Fig. 8a, right) with reduced hinge length is chosen, which has a similar torsional stiffness as the CRCJ. Conventional compliant hinges with thin flexible structure exhibit low torsional stiffness. CRCJ, however, is less sensitive against torsion due to the bulky cylinder cams and the flexure straps connecting in between. In addition, FEA results of the force bending angle relation of CRCJ were also compared with the experimental measurements.

#### Experiment 1: bending motion

Figure 8c illustrates the experiment set-up for the bending motion test. A measurement equipment to actuate the CRCJ with a single cable using a pulley system is designed. The actuation pulley has a radius of 6 mm, and the actuation cable is connected to a digital force sensor (DIGIFORCE$\textregistered$ 9310, burster präzisionsmesstechnik gmbh & co kg, Gernsbach Germany). The single actuation (see Fig. 8b, c, red) tendon for the joint is assembled with a pulley underneath the pulley system. Both pulleys are printed on a monolithic structure. On the top surface of the joint, the tendon is fixed on both sides. The relationship between the cable force $F_{\mathrm{c}}$ and actuation force of on the top surface of the manipulator $F_{\mathrm{a}}$ can thus be formulated as:

6 $$\begin{aligned} F_{{\mathrm{c}}} = \kappa \frac{F_{\mathrm{a}}c}{r} . \end{aligned}$$

The factor $\kappa$ is introduced as a factor to capture the effects of friction in the force transmission, e.g. in the pulley system or in the tendon guides due to manufacturing inaccuracy. The bending angle in this experiment is measured using the Navigation camera (Polaris Vicra, NDI, 103 Randall Drive, Waterloo, Ontario, Canada). For this purpose, a form-fitting tracker is attached to the measurement set-up and two markers are mounted on the top of the testing unit, defining the boundary of the top surface (cf. Fig 8a). The position of both markers **p** in the coordinate system of tracker $CS_{\mathrm{tr}}$ can be derived from:

7 $$\begin{aligned} ^{\mathrm{tr}} \mathbf{p} =(^{\mathrm{cam}} \mathbf{T} _{\mathrm{tr}})^{\mathrm{T}} \,\, ^{\mathrm{cam}} \mathbf{p} . \end{aligned}$$

In (7), $^{\mathrm{cam}} \mathbf{T} _{\mathrm{tr}}$ and $^{\mathrm{cam}} \mathbf{p}$ represent the homogeneous transformation matrix from camera to tracker coordinate system and the position of marker points in the camera coordinate system, respectively, which are given by the navigation system. The bending angle can be derived using geometry relationships between the initial position and the current position of both markers after the joint is deformed under load. For the bending motion experiment, the relationship between force and bending angle was determined using 3 samples of each joint type. In this work, we define the bending stiffness *K* as the quotient of the applied cable force $F_{\mathrm{c}}$ and the bending angle $\theta$

8 $$\begin{aligned} K = \frac{F_{\mathrm{c}}}{\theta } . \end{aligned}$$

Linear correlation of force and bending angle can be shown in the measurement data in Fig. 9a for all joint types. Therefore, linear regression was applied to fit the measurement data of all 9 measurement series. The polynomial coefficients of each sample, which contain the slope $P_1$ and y-intercept $P_0$ are summarized in Table 2.

**Table 2:** Polynomial coefficients $P_0$ and $P_1$ from linear regression of measurement data of force bending angle

Sample No.	CRCJ		LEAF-TYPE 1		LEAF-TYPE 2	
	$P_0$	$P_1$	$P_0$	$P_1$	$P_0$	$P_1$
1	25.59	6.36	50.37	6.64	22.57	5.33
2	23.84	6.86	48.44	10.72	21.63	2.32
3	25.72	12.18	48.75	10.86	21.93	0.44
Mean	25.05	8.48	49.19	9.41	22.04	2.70

**Fig. 10:**
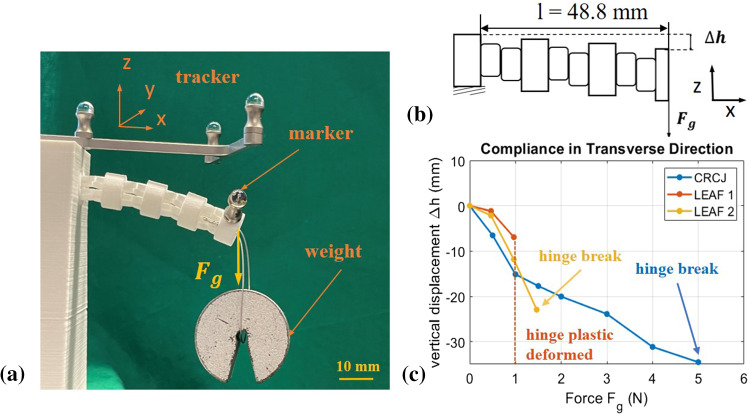
Experiment set-up for compliance test in transverse direction. **a** Schematics of the compliance test in transverse direction: the tip of the joints are loaded with weights. The length of free segment is 48.8 mm. **b** Experiment set-up of the compliance test in transverse direction. The deflection of the joint in transverse direction $\Delta h$ is measured. **c** Experiment measurement data of loading in transverse direction ${F}_{\mathrm{g}}$ in relation to vertical displacement $\Delta h$

The mean values of both polynomial coefficients were determined for each joint type. These were used to plot the force bending angle relation of each joint type in Fig. 9b. Based on the deformation comparison between FEA and experiment, $\kappa = 2.13$ in (6) was determined. While the leaf-type joint 1 has the lowest bending stiffness, leaf-type 2 has the highest. Leaf-type joint reaches the threshold of test break already at about 3.6 N. Due to the high intrinsic elasticity and the contact restriction of the spacing discs, leaf-type joint 2 can only reach a maximum bending angle of around $\theta =160^{\circ }$ as shown in Fig. 9. The bending stiffness of CRCJ is slightly lower than the bending stiffness of leaf-type joint 2. This shows the higher flexibility of CRCJ in the bending plane compared to conventional leaf-type joint of similar torsional stiffness. In addition, CRCJ can even surpass $180^{\circ }$ bending angle thanks to its rolling-contact mechanism. During this experiment, cable slack was not observed in CRCJ but in both leaf-type joints, which also reflects in the lower bending stiffness of the CRCJ compared to leaf-type 2 due to better force transmission. Result from the FEM analysis also shows a good agreement with the experimental data, which validates the modelling of nonlinear cable force given in (4) and (5).

#### Experiment 2: compliance in transverse direction

The compliance test in transverse direction was conducted to explore the flexibility of the compliant joints in the plane perpendicular to the bending plane. The experiment set-up is shown in Fig. 10a. A high compliant motion can be advantageous for applications in surgical procedures to prevent tissue injury [13]. The experiment set-up is schematically depicted in Fig. 10b. The joints are rotated $90^{\circ }$ around the axis to use gravity force as the transverse force ${F}_{\mathrm{g}}$. Weights are then added to increase transverse loading. The length of free segment is 48.8 mm. As shown in Fig. 10c, both leaf-type hinges show very little compliance in transverse direction. While the second broke at ${F}_{\mathrm{g}}=1.46\, \hbox {N}$, the first can hardly resist any transverse force and the hinges undergo plastic deformation after applying only ${F}_{\mathrm{g}}=0.96\, \hbox {N}$. However, the compliant motion of CRCJ in transverse direction can be well demonstrated in this experiment, in which the vertical displacement shows a linear relationship with the applied force. With a deformation $\Delta h$ of over 30 mm, the joint structure was still able to resist the load and to recover to its initial state after removing the transverse load. The CRCJ first broke after ${F}_{\mathrm{g}}=5\, \hbox {N}$ was applied to the tip.

#### Experiment 3: compliant motion

**Fig. 11:**
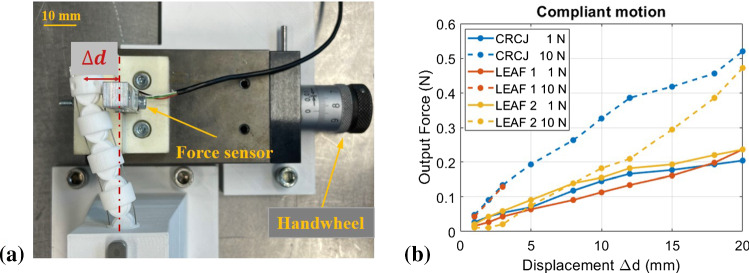
Experiment set-up for compliant motion test. **a** Testing joints with constant cable tension of 1 N and 10 N were bent under an external load and **b** relationship between the lateral displacement and force exerted by the joint

For medical application, continuum manipulators are required to have sufficient flexibility to adapt to the shape of the cavity and to prevent tissue injury. The compliant motion test is aimed to characterize the stiffness of the manipulator structure against external force in the bending plane. Constant tension of 1 N and 10 N were applied to all three joint types. The experiment set-up is depicted in Fig. 11. The bending motion of the CRCJ under environment loads can also be observed. In this experiment, the force sensor simulates the inner wall of an organ or other tissues. The displacement can be controlled by the handwheel of the coordinate table. The input displacement was set from 0 to 20 mm.

First, all three joints showed similar supportable external force under cable tension of 1N on the tip of the manipulator at about 0.2 N with lateral displacement of 20 mm. When the actuation cables were tightened to 10 N, the maximum output force in CRCJ and leaf-type 2 also increased to about 0.5 N with a factor of 2.5. The experiment of leaf-type 1 with tendon tension at 10 N was interrupted since buckling of the flexure hinges was observed. The forces sensor also showed an output force of 0 N, which indicates that the flexure hinges lose their stability. While this problem may also occur at larger cable tension in joint leaf-type 2 due to similar hinge design, the compliant motion of CRCJ will not be affected by this problem because of rolling contact between the flexure straps and cylinder cams. With higher cable tension, the joint gap *gapb*, which is designed intentionally as described in “Geometry generation and fabrication” section, will reduce. In General, the supportable force exerted by the manipulator remains very low in range below 1 N, which is less than 20 % of the required force for surgical operations [24]. Compared to the non-monolithic contact rolling joint design in [13] with a length of 55 mm, our CRCJ structure exhibits similar behaviours of compliant motion.

## Application

**Fig. 12:**
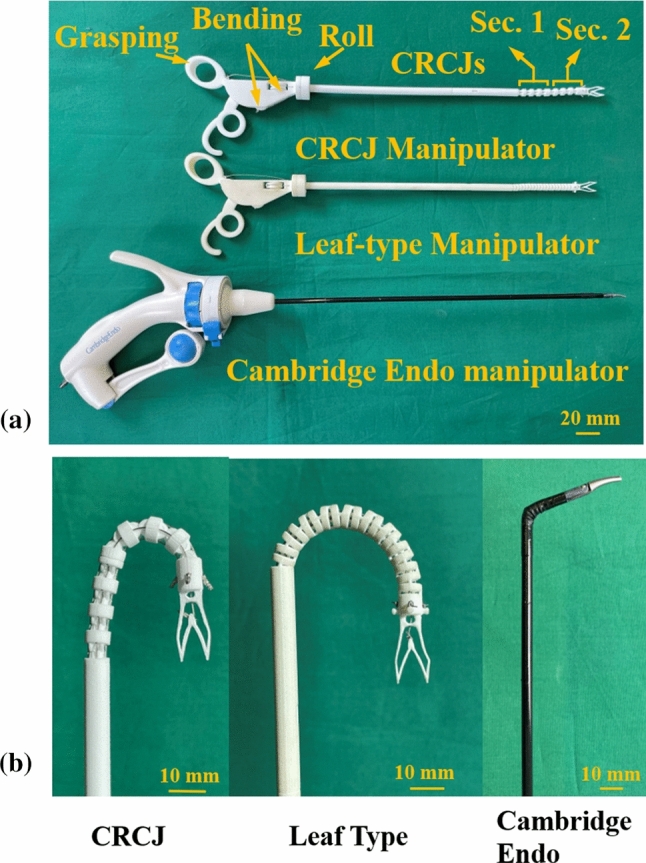
Comparison of SLS-manufactured manipulator prototype using CRCJ with leaf-type manipulator and Cambridge Endo manipulator. **a** Top: SLS-manufactured prototype of a 3-DOF handheld laparoscopic manipulator using CRCJ. Mid: previous handheld manipulator using leaf-type joints. Bottom: Cambridge Endo laparoscopic manipulator. **b** The maximum bending angle of abovementioned manipulators. CRCJ can achieve the bending angle of $180^{\circ }$ and has the smallest bending radius

Using the proposed CRCJ structure, as well as the geometry design and fabrication method in “Methods” section, the prototype of a 3-DOF (two bending and a roll motion) handheld laparoscopic manipulator was implemented. Figure 12a provides the structure of the prototype. The CRCJ manipulator employs two CRCJ sections with each section consisting of 4 joints, that are located at the distal tip of the manipulator. The two CRCJ sections are rotated $90^{\circ }$ around the axis so that bending motion can be performed in two perpendicular planes using the handle. The rolling motion is further provided by the shaft. Grasping is also integrated in the prototype by the automatic designed compliant forceps using topology optimization in [25]. The shaft and the flexible sections of the manipulator have a diameter of 7 mm. Including the handle, a total of 4 parts are required to assemble the prototype. Compared to the Cambridge Endo manipulator, which is shown on the bottom of Fig. 12a, the part count of our design can be reduced thanks to the use of compliant mechanism instead of traditional rigid mechanism for both the joints and forceps. In addition, the forceps and the second CRCJ section are integrated and fabricated in one part thanks to the SLS-technology. The reduced part count alleviates the assembly and sterilization effort. The dexterity and flexibility of the CRCJ manipulator are further demonstrated in Fig. 12b. All handheld manipulators shown in Fig. 12 are actuated to the maximum bending angle. The proposed CRCJ and our previously designed manipulator based on leaf-type hinges can both achieve $180^{\circ }$ bending angle, while the bending angle of Cambridge Endo is less than $90^{\circ }$. Among three manipulators, the CRCJ manipulator has the smallest bending radius.

**Fig. 13:**
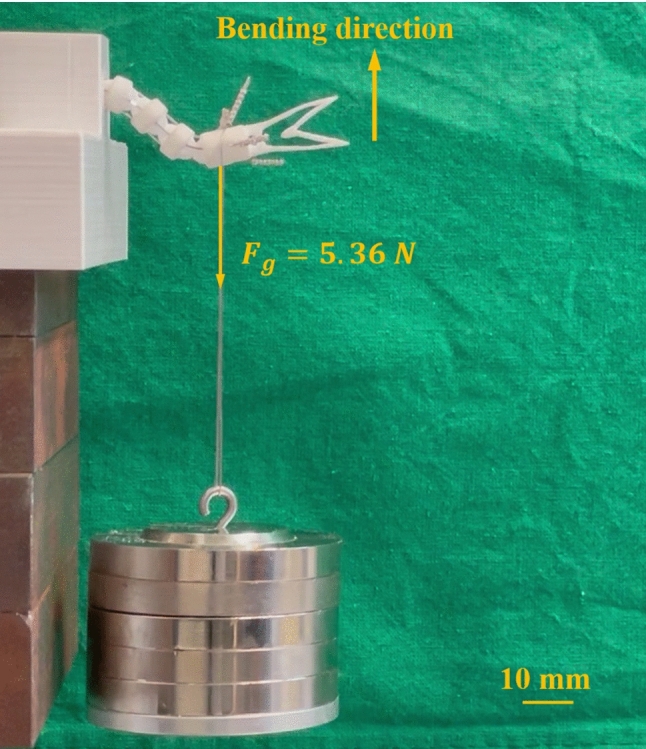
Lifting of weights (546.5 g) attached to the tip of CRCJ manipulator prototype

## Discussion

In the course of the development of additive manufacturing, complex structures that are difficult to realize using conventional reductive manufacturing techniques are becoming easier and less expensive to fabricate. Complex compliant mechanisms such as the CRCJ proposed in this work can be easily realized and fabricated in a monolithic structure by powder-based additive manufacturing techniques. With reduced part count, assembly and sterilization process can be simplified. However, the design process should be optimized and adjusted due to the anisotropic material properties from additive manufacturing process, which is especially critical for joint structures which are utilized for the design of steerable manipulator structures. Furthermore, complex compliant structures usually embody complex motion behaviour, which may require new modelling method such as nonlinear FEM.

In the present work, a preliminary prototype of a handheld manipulator for laparoscopic surgery was realized using the proposed CRCJ mechanism which is fabricated using PA2200 with SLS. For real laparoscopic surgery such as the laparoscopic sigmoid resection (LSR), an estimated mean force of 4.79 N was identified based on an experiment in a porcine model in [24]. The proposed device using monolithic CRCJ section can successfully apply a force of about 5.36 N in the operation direction, which corresponds to 546.5 g attached to the instrument’s tip, as shown in Fig. 13. However, it remains challenging to reduce the applied operating forces of the manipulator. This could be achieved through a pulley system or enhanced force transmission using double wires Bowden cables as described in [26]. In order to improve the stiffness of the manipulator, sheaths for tendons and an outer sheath can be applied in the design. The latter can also prevent the blood or tissue contact with the monolithic CRCJ structure, in which the gap between flexure straps and cylinder cams can cause critical issues during surgery. Using metallic material can further improve the stiffness and the repeat accuracy of the proposed manipulator [11]. Metallic additive manufacturing can be used to fabricate application-oriented prototype. An increase in stiffness will usually cause a loss of operability. Thus, design improvements can be achieved by optimizing the proposed manipulator regarding the operation-specific required manipulation force and flexibility.

In addition, design concept using CRCJ that has a larger centre tool lumen is required so that it can be integrated in robot-assisted surgery. Nonetheless, CRCJ reveals promising mechanical properties including large bending motion, flexibility and compliant motion in both the bending plane and transverse direction, as shown in the experiments in “Simulation and experiments” section. These make CRCJ suitable for medical applications, in which dexterity and compliance are both relevant.

## Conclusion

In this work, the design of a novel monolithic tendon-driven manipulator structure using CRCJ is proposed, which combines the advantages of both compliant mechanism and rolling-contact mechanism. Both FEA and experiments have been conducted to analyse the bending and compliant motion of the proposed structure. Experimental results reveal that the CRCJ has higher flexibility, compliance and a smaller bending radius compared to conventional compliant leaf-type hinges. A preliminary prototype of a handheld laparoscopic manipulator fabricated by SLS demonstrates the capability of the CRCJ for steerable medical devices. Since the FEA shows good agreement with the experimental results, further design improvements based on FEA can be made. Application-oriented prototypes using metallic materials and the integration of CRCJ into robot-assisted MIS with consideration of anatomical and surgical requirements would be a natural next step.

## Supplementary Information

Below is the link to the electronic supplementary material.

Supplementary material 1 (mp4 7354 KB)
